# A Qualitative Study on Knowledge, Attitude, and Practice of Nursing Students in the Early Stage of the COVID-19 Epidemic and Inspiration for Nursing Education in Mainland China

**DOI:** 10.3389/fpubh.2022.845588

**Published:** 2022-03-14

**Authors:** Houxiu Zhou, Rongrong Zhao, Yanni Yang

**Affiliations:** Department of Nursing, Army Medical University (Third Military Medical University), Chongqing, China

**Keywords:** COVID-19 epidemic, nursing students, KAP, qualitative study, training

## Abstract

**Background:**

At the beginning of the COVID-19 outbreak, there was a lack of sufficient nursing experience for pneumonia caused by COVID-19. All nursing decisions had to be innovatively made and measures taken by nurses using their existing knowledge and skills. This required nurses to have a solid theoretical understanding of infectious diseases and epidemiology, evidence-based solid practice skills, and problem-solving skills. The COVID outbreak reminded undergraduates to master relevant knowledge and abilities during school study.

**Methods:**

Qualitative research on knowledge, attitude, and practice (KAP) of the COVID-19 epidemic was conducted using semi-structured interviews among sophomore nursing students in the university. Based on the characteristics of the KAP of nursing students, we analyzed the deficiencies of the knowledge and ability to deal with large-scale public health emergencies in the second-year nursing education.

**Results:**

A total of 12 subject headings and 41 sublevel headings were identified from three aspects of KAP. The subject headings included knowledge aspect (the origin of the disease, the route of transmission, main symptoms, the epidemiological characteristics of the disease, scientific cognition of information sources), attitude aspect (different emotional experiences, a certain degree of influence, different views on the development trend of the epidemic, support the government's prevention and control strategies), and behavior aspect (do an excellent job in self-protection, help family members to protect, and participate in social anti-epidemic actions). According to this analysis, second-year nursing students have three deficiencies in dealing with large-scale public health emergencies: knowledge of infectious diseases and epidemiology, evidence-based practice skills, and problem- solving ability.

**Conclusion:**

When students start nursing professional courses, the knowledge of infectious diseases and epidemiology, training of evidence-based practice skills, and problem-solving ability should be strengthened to improve the ability of nursing undergraduates to respond to large-scale public health emergencies after entering the workplace.

## Introduction

According to the training goal for undergraduate nursing students stipulated by the China Ministry of Education, undergraduate nursing students are required to “have the capability of engaging in clinical nursing, preventive health care, nursing management, nursing research, and other work in the field of nursing” (1). This requires nursing students to master the relevant theories and skills of the nursing discipline and have the ability to deal with nursing emergencies and solve professional nursing problems, which is especially important in the handling of public health emergencies. In previous public health emergencies, nurses have been an indispensable part of the rescue force, and their rescue tasks involve dealing with a large number of emergencies and solving various emergency problems that have never been encountered before. There are no authoritative and ready-made guidelines or methods to solve these problems. Instead, nurses must use their evidence-based practice skills and problem-solving skills to make scientific decisions quickly. The sudden outbreak of the COVID-19 epidemic in 2019 once again tested nurses' abilities.

As the main force of the anti-epidemic team, since the outbreak of COVID-19 in China in December 2019, China has sent a total of 42,000 elite medical forces to support Hubei (the epicenter of the outbreak), among them 28,600 were nurses, accounting for 68% of the medical personnel (2). Nurses have played an essential role in the fight against the epidemic. They have completed the rescue mission through their excellent professional skills and deep humanistic care capabilities (3).

Since the epidemic of COVID-19, researchers have published many articles in the field of nursing, accumulating valuable information for the clinical practice of nursing in responding to large-scale public health emergencies. However, few reports are on the importance and challenges of the COVID-19 epidemic in nursing education. There is no literature on the relevant deficiencies in current undergraduate nursing education. The COVID epidemic poses a unique challenge for training the next generation of nurses (4). In the early stage of the epidemic, the medical personnel who participated in the Wuhan rescue mission deeply felt that there was still a lack of systematic understanding of public health emergencies and emphasized the training of the health system in the prevention and control of public health events such as sudden infectious disease outbreaks (5). Medical schools are the best place for medical personnel to receive vocational training. As future nurses, nursing students must receive relevant training at the undergraduate level. The epidemic that occurred should be used as an opportunity to improve the education program of future health professionals and, to a greater extent, incorporate the necessary abilities to improve knowledge and attitudes, confidence, and social and political responsibility when faced with epidemic outbreaks (6).

In China, most of the supporting nurses currently involved in front-line epidemic prevention had deep feelings and experiences during the SARS epidemic 17 years ago, which sowed the seeds for their devotion to the nursing career when they were nurses. During the outbreak of SARS, nursing students were finally able to respond calmly to that public health emergency after experiencing a complex process of extreme fear and anxiety, trying to escape reality, overreacting, and managing the changes (7). Nursing educators used the anti-epidemic situation and factual information to conduct moral education for nursing students and achieved apparent results (8). The epidemic events and the contributions of nursing staff during the SARS epidemic provided nursing students with a deeper understanding and knowledge of their major. They hoped to improve their quality and become excellent nurses to serve people's health in the future better (9). Seventeen years later, as mature nurses working in various fields, these students reencountered similar public health emergencies and were able to bravely and calmly dedicate themselves to fighting the epidemic. This ability may be related to the education and experience during the SARS epidemic. Currently, nursing students are experiencing a more serious public health event than SARS. As nurse educators, we should use this event to provide better vocational education for nursing students. According to the social responsibilities after graduation, besides teaching relevant knowledge and skills, we must also cultivate their key ability to deal with sudden epidemics. Nurses' abilities depend on the training in previous education and daily work. On the other hand, in public health emergencies, the knowledge, attitude, and practice (KAP) of undergraduate nursing students can better reflect their level of professional knowledge and ability and reflect the problems of current nursing education in training students in dealing with public health incidents, thus improving the effectiveness of nursing education.

KAP was first proposed by the British health educator John Coster, who believes that a person's cognitive process include three continuous domains: knowledge, attitude, and practice/behavior. Knowledge is the foundation, attitude is the driving force, and behavior is the goal. Based on understanding of health care knowledge and information, people establish positive and correct beliefs and attitudes, use this as a driving force, and then take the initiative to form healthy behaviors or changes. The theory of knowledge, belief, and behavior is used in nursing education to change the behavior of nursing students. It is relatively mature. It includes three continuous processes of knowledge teaching, belief cultivation, and behavioral norms to comprehensively improve the post-education competency of nursing students (10, 11). When KAP theory is applied to nursing teaching, it is necessary to assess the knowledge, attitude, and skills that nursing students possess on specific teaching topics, analyze the deficiencies, and design targeted teaching improvement plans. The second-year nursing undergraduate students at a nursing school have learned basic disciplines such as microbiology and epidemiology but have not yet started to learn nursing professional knowledge. At this time, they are studying their KAP on COVID to understand their response to new, unknown, sudden viruses. The response to the large-scale public health events can be educated in the future teaching of professional nursing courses. Due to the sudden occurrence of COVID-19, there is no relevant literature on the impact of the epidemic on nursing education as a basis and reference in the data collection stage.

The study aimed to answer two questions: (1) what are the characteristics of knowledge, attitude, and practice of undergraduate nursing students in coping with large public health events? and (2) what are the implications of the characteristics for the current education of undergraduate nursing students to respond to and participate in major public health incidents? Currently, the world is still in a COVID pandemic. The findings of this study may provide valuable information for nursing education in China and other countries.

## Methods

This study adopted a qualitative approach to phenomenological methods. The purpose of the phenomenological interviews was to obtain detailed and multidimensional qualitative information about the participants' views, knowledge, experiences, feelings, attitudes, and habits concerning specific themes (12).

### Participants

Due to the sudden outbreak of COVID-19 during winter vacation, per the national prevention and control instructions, everyone must be isolated at home. It was inconvenient for teachers and students in other schools to contact. This study adopted the convenience sampling method and selected the students in the researchers' teaching class, with information saturation as the principle to determine the sample size. From 10 February to 15 February 2020, second-year nursing undergraduates from a medical university in Chongqing were selected as participants by purposive sampling. The inclusion criteria were the following: (1) full-time sophomore nursing majors, (2) staying at home for more than 2 weeks, (3) having good communication and presentation skills, willingness to participate in this study, and signing the informed consent form. The exclusion criteria were as follows: inability to communicate by telephone for various reasons (for example, poor health or home without good phone coverage).

### Ethics Consideration

This study was approved by the university ethics committee (ethic approval number: 2020 No. 006-02) and obtained informed consent from all participants. Participants were volunteers, and they were ensured that the research data would be kept strictly confidential and presented anonymously; Furthermore, participants were allowed to withdraw at any time (13).

### Data Collection

Data collection was conducted using semi-structured interviews for qualitative research. Based on the current situation of home isolation during the COVID-19 epidemic, the interviews were conducted by telephone. The outline of this study was agreed upon by three members of the research team. The main questions included the following.

1. What is your current knowledge of COVID-19?
2. What do you think of COVID-19?
3. What have you done about COVID-19?
4. What else do you think you should do if conditions permit?
5. What is the biggest problem you have encountered during the COVID-19 pandemic?

All interviews were completed and recorded by the same researcher (principal researcher, Doctor of Nursing). Each subject answered all the questions in the outline for 30–45 min. Before the interview, an appointment was made to confirm that the interviewee was in a quiet and uninterrupted room with enough time. The researcher avoided using judgmental, forgiving, or negative statements and attitudes during the interviews.

### Data Analysis

A qualitative method was adopted for the data analysis. After each interview, the transcription of the recorded material was completed, and the principal researcher used the thematic framework analysis method to analyze and summarize the data. The steps were as follows: (1) familiarity: read the text repeatedly to become familiar with the qualitative data; (2) encoding: use the open coding method to analyze the data line by line and record the essential words and phrases (units of meaning) in the margins of the material; (3) integration: describe each significant unit of meaning in a descriptive code and integrate codes with the same meaning (14). After the analysis, two other researchers (Ph.D. and Master's of Nursing Science) who were familiar with the telephone recordings conducted a peer review to ensure the reliability of the analysis results.

## Results

### Basic Information of Participants

Fifteen second-year undergraduate nursing students were included in this study, including 12 female students and three male students. Respondents were 19–22 years old, with an average age of 20.33 ± 0.82 years. The participants were numbered N1–N15; N1–N12 were female, and N13–N15 were male. All participants have completed the courses of pathology, microbiology, epidemiology, and other public subjects and passed the examinations, and have a basic understanding of the common characteristics of the pathogenesis and transmission of viruses and other microorganisms. All participants had not yet started their nursing professional courses.

### Interview Results

#### The Characteristics of the Interviewees on KAP in Response to the COVID-19 Epidemic

Twelve main themes and 41 sub-themes were identified (Table 1).

**Table 1:** Main themes and sub-themes from the study.

**KAP**	**Main themes**	**Sub-themes**	**Participants**
Knowledge	The origin of the disease	Wild animals	N1, N2, N3, N4, N5, N6, N7, N8, N9, N10, N11, N12, N13, N14, N15
	The route of transmission	Droplet transmission	N3, N4, N7, N8, N9, N15
		Contact transmission	N3, N4, N7, N8, N10, N12
		Aerosol transmission	N7, N10, N11
		Air transmission	N6
	Main symptoms	Fever, fatigue, dry cough	N9, N10, N12, N14, N15
		Difficulty breathing	N9, N10, N12, N15
		Respiratory distress	N10
		Respiratory failure	N9
	The epidemiological characteristics of the disease	Spread fast	N1, N7
		The population is generally susceptible	N1, N4, N7, N10
		Susceptible groups are the elderly and those with chronic underlying diseases	N8
	Scientific cognition of information sources	CCTV News	N1, N3, N4, N5, N6, N8, N10, N11, N14
		Weibo	N1, N2, N5, N7, N8, N10, N11
		Expert interview	N7, N11, N14
		WeChat	N6, N8, N13
		Brochure	N1, N13
		Baidu Encyclopedia	N8
Attitude	Different emotional experiences	Panic and fear	N1, N2, N6
		Touched	N11
	Consider themselves to be affected to some extent	Inconvenience	N1, N2, N7, N8, N11, N14
		Plans are disrupted	N3, N4, N5, N6
		Socially restricted	N9, N10, N12, N15
		Normal learning is limited	N13
	Different views on the development trend of the epidemic	Believe the epidemic will be brought under control soon	N3, N4, N5, N6, N9, N11
		Believe that the development trend of the epidemic is uncertain	N13
		Believe that the epidemic will recur for a long time	N15
	Support the government's prevention and control strategy	Willing to listen to the call of the state and implement official requirements	N1, N3, N4, N5, N6, N7, N8, N9, N10, N11, N12, N13, N15
		Wanting to participate the fight against the epidemic	N3, N4, N6, N7, N9, N11, N13, N15
Practice	Self-protection	Try not to go out	N1, N4, N7, N8, N11, N12, N13, N15
		Wear a mask when going out	N5, N7, N8, N14
		Avoid going to crowded places	N1, N5, N8, N15
		wash hands frequently	N7
		Actively adapt to isolation	N4, N5, N6, N7, N10, N12, N13
	Help family members protect	Educate family members about protection	N1, N2, N3, N4, N6, N8
		Ask family members to stay home	N3, N4, N5, N14, N15
		Disinfect household items and environment	N4, N14
		Monitor the health of family members	N6
	Participate in social anti-epidemic actions	Keeping an eye on the epidemic	N1, N3, N4, N10, N11, N12, N14, N15
		Promoting correct anti-epidemic knowledge through WeChat	N1, N4
		Donate	N1
		Volunteer	N4

### Sophomore Nursing Students' Knowledge of COVID-19

#### Knowledge of the Origin of the Disease

The 15 participants all mentioned that the virus came from wild animals. N1: “Many wild animals are the host of this novel coronavirus, such as civets, bats, rodents, and badgers. Therefore, it is speculated that the cause of this new pneumonia is transmission between wild animals and humans.” N12: “The natural host of the virus is bats. The intermediate host has not yet been identified but is now thought to be some semi-wild mammal that can be fed on a large scale.” In the early stage of data collection and outbreak of this study, because the initially identified outbreak occurred in the wild animal market, the mainstream view at that time was that it originated from wild animals. However, this statement did not appear in the China Center for Disease Control (CDC) or the World Health Organization (WHO) guidelines.

#### Knowledge of the Route of Transmission

Respondents' views: The virus can enter the human body through contact, droplet, aerosol, and air transmissions. N3: “It transmits mainly by respiratory droplets, but also by contact.” N6: “…is mainly airborne.” N10: “COVID-19 is transmitted mainly by aerosol and contact.” The “Guidelines for Public Protection of Pneumonia Infected by Novel Coronavirus” issued by China CDC on 31 January 2020 (15) mentioned transmission routes include direct transmission, aerosol transmission, and contact transmission. The “WHO 2019 Novel Coronavirus Strategic Preparedness and Response Plan” (16) issued on 10 February 2020 mentioned that the transmission routes include droplet transmission, contact transmission, and transmission between people and objects.

#### Knowledge of the Main Symptoms

The main symptoms mentioned by the respondents included fever, fatigue, dry cough, dyspnea, respiratory distress, and respiratory failure. N9: “The main clinical manifestations of patients are fever, fatigue, and dry cough, and some severe patients may have difficulty breathing or even respiratory failure.” N10: “Severe cases rapidly progress to symptoms such as acute respiratory distress syndrome.” The symptoms mentioned by the respondents were broadly consistent with the guidelines issued by the China CDC on 31 January 2020 (15).

#### Knowledge of the Epidemiological Characteristics of the Disease

The epidemiological characteristics mentioned by the majority of the respondents include rapid spread and general susceptibility of the population. N1: “It has rapidly spread with large susceptible populations and relatively low mortality.” N4: “The population is generally susceptible, and the elderly with chronic underlying diseases can progress more rapidly, and have greater severity and poorer prognosis after infection.” N8: “The susceptible population is the elderly and those with chronic underlying diseases.” Some respondents also believed that susceptible groups were the elderly and those with chronic underlying diseases. The guidelines issued by the China CDC stated that “deaths are more common in elderly patients and those with underlying diseases” (15).

### Respondents' Perceptions of High-Level Sources of Relevant Literature Evidence

Unscientific information is an important cause of panic among people. In the early days of the COVID-19 outbreak, there were many sources of information. Respondents considered more scientific sources of information to include China Central Television, Weibo, expert interviews, WeChat, brochures, and the Baidu Encyclopedia. N1: “The ‘New Coronavirus Pneumonia Prevention Manual' published by Hubei Science and Technology Press, as well as some information from CCTV News and Weibo, are highly credible because they are all from the official government.” N8: “News, Baidu Encyclopedia, because since it can be published in the public media, it must have been tested, and it is the research result of experts.” None of the interviewees mentioned the latest guidance from China CDC, WHO, and the US CDC.

### Views and Attitudes of Sophomore Nursing Students Toward the COVID-19 Epidemic Event

#### Different Emotional Experiences

The sudden outbreak of a major epidemic resulted in the suspension of work, school and being at home. All interviewees were involved. Medical colleagues stepped forward and risked their lives to fight the epidemic. Community managers actively promoted various forms of propaganda and maintained order. They encouraged each other and overcame difficulties together, leading to different emotional experiences of respondents, including panic and emotion. N1: “Many people who are not at the center of the outbreak, especially those with inaccurate information, spend their days in a state of panic and relaxation” (referring to people who were not in Wuhan at that time and were scared by the outbreak but were unwilling to actively protect against it). N2: “I was terrified for 14 days because I had worked in a store and had come into contact with all kinds of people. I was afraid there might be a virus lurking in my body, especially when my cough started, but fortunately, I was not infected.” N6: “I feel like I am actually on the hook; I am going through a period of historical catastrophe and have a sense of participation.” N11: “Cry for the people who stick to their posts on this land and cheer for the medical staff and infrastructure workers who rushed to Wuhan.”

### Consider Themselves to Be Affected to Some Extent

In the state of home isolation, the respondents believe that the impact includes inconvenience in life, disrupted plans, social restrictions, and normal learning restrictions. N4: “Not being able to go out caused me to cancel the winter vacation plans I had made in advance. I also cannot begin the next semester (return to school) normally.” N10: “The biggest problem is that I cannot go out. I am bored staying home for so long.” N11: “Cannot go out, stores are closed, life is not.” N15: “I cannot go out, and I cannot see my friends and relatives. After going to college, I have had very few opportunities to see my friends and relatives, and the Spring Festival should have been a vacation to visit others all over the world.”

### Different Views on the Development Trend of the Epidemic

Some participants believed that the epidemic could be controlled after a period of time if the prevention and control system was strictly observed throughout the country. N4: “The number of people infected will continue to increase in the short term, but the number of people cured will increase more and faster.” However, some participants remained concerned. N12: “The number of newly infected people is decreasing in other areas except Hubei and other key epidemic areas. The epidemic is relatively easy to control if the source of infection is controlled and situation in the key epidemic areas is still severe.” N15: “While various measures have been taken to prevent it, it has only slowed the progress of the epidemic. If there is no specific drug, I think the epidemic may be repeated.” Some said the trend was still unclear: N13: “Future trends remain to be seen.”

### Support the Government's Prevention and Control Strategy

Most of the students are willing to listen to the state's call and implement official requirements. N6: “We must respond to the call of our country and stay home.” N10: “I think it is necessary to stay home, not only to prevent myself from being infected but also to make a small contribution to the country's early recovery.” N13: “I think that we as citizens should support the policy of our country in the face of the disaster.” Participants were willing to take action to prevent the epidemic, such as making epidemic prevention propaganda for residents and helping the community in screening. However, because they were required to be isolated at home and there was a severe shortage of supplies, these things were just wishes and were not implemented. N3: “If conditions permit, I may also go to the front line to do what I can, such as disinfecting the community and monitor the temperature of suspected people every day.” N4: “(I want to) participate in the local volunteer association and join the traffic police at the county highway intersection for screening.” N10: “If the conditions permit, I hope to go into the community and the countryside. Many people do not understand the severity of the outbreak at an early stage. If we can publicize and improve the awareness of residents and the awareness of the new coronavirus, we can reduce infection.” N15: “If conditions permit, I really hope to go to the front line to fight against the epidemic, even as a triage person is acceptable to me.”

### The Practice of Sophomore Nursing Students in the COVID-19 Epidemic Event

#### Self-Protection

The interviewees said they should protect themselves first, including staying home as much as possible, wearing a mask when out, avoiding crowded places, washing hands frequently, and actively adapting to the isolation state. N5: “I basically went to the store to buy a mask as soon as possible…I try not to go out, wear a mask when I go out…I learn English on my mobile phone at home, watch open nursing classes, and learn some dances with my mother. These.” N7: “Stay at home, go out as little as possible, wear a mask when going out, wash your hands frequently, etc., and do personal protection.”

### Help Family Members Protect

Respondents used their professional knowledge to help their family members protect, including preaching protection knowledge to family members, asking family members to go out less often; disinfecting household items and environments; and monitoring their family members' physical conditions. N2: “I used my knowledge learned last semester to help my family verify some misunderstandings about the virus, but there are still some areas that I do not know enough about. I will go online to learn about them.” N4: “Study the knowledge and protective measures of the new coronavirus in the family… Do a good job in household disinfection.” N6: “As a person with some medical knowledge, I also acted as a family doctor, publicizing the hazards and preventive measures of COVID-19 in real-time and monitoring the health of my family members in order to protect my family and make a small contribution to the country…….and monitor the health of family members in a timely manner.”

### Participate in Social Anti-epidemic Actions

Respondents have participated in various anti-epidemic actions, including keeping an eye on the epidemic, promoting correct anti-epidemic knowledge through WeChat, donating money, and serving as volunteers. N1: “Donate to affected areas… Continue to pay attention to the real-time epidemic situation and learn some new developments reported.” N4: “(I) understand the effective protection measures of COVID-19 and pay attention to the situation of the domestic epidemic every day……Participate in the local volunteer association, and conduct investigations with the traffic police at the expressway intersection in the county (later terminated because the school uniformly conducts online classes).” N9: “I keep an eye on the news.”

### Enlightenment of the KAP Characteristics of Second-Year Nursing Undergraduates Dealing With COVID-19 on the Teaching of Nursing Professional Courses

#### Knowledge of Epidemiology and Epidemiology Needs to Be Strengthened

Participants realized that the disease originated from wild animals could be transmitted by droplets through the respiratory tract, aerosols, and contact; its transmission speed was fast with general susceptibility. They also knew the number of infected persons on the day the interview was conducted and to stay home and wear a mask to protect themselves from infection. Their knowledge in these fields was mainly correct based on the official reports on the novel coronavirus. However, this information was known to the general public and promoted by various news media and articles. As medical majors with a background in microbiology, the participants lacked exploration and reflection on other virus characteristics. For example, participants could not present corresponding views and opinions on reproduction, transmission, and inactivation conditions, and other prevention and control methods for the coronavirus epidemic event, showing that participants have not yet reached a satisfactory level of knowledge of related epidemics, which is consistent with the results of a large sample of domestic medical students (17).

### Capacity for Evidence-Based Practice Needs to Be Strengthened

The sources of information considered by the participants to be more scientific include CCTV news, Weibo, expert interviews, WeChat, brochures, and Baidu Encyclopedia, which shows that the participants have not yet mastered the skills of how to judge the strength of clinical practice evidence and how to obtain high-level evidence quickly. From an evidence-based practice perspective, these are not high-level evidence, and there is even a lot of misinformation spreading through these channels. Although it was in the early stage of the epidemic, official prevention and control guidelines have been published, and more and more academic articles have been published. Unfortunately, the participants ignored the information. None of the participants mentioned academic papers published in professional journals. This shows that participants lacked the awareness and ability to apply evidence-based practice in problem-solving, as reflected in the participants' insufficient ability to collect professional information, analyze and evaluate data, and perform critical analysis.

### Problem-Solving Skills Need to Be Strengthened

Most of the participants proposed that they wanted to devote themselves to the actual act of fighting the epidemic and do something within their capacity. However, only one participant acted by joining a group of volunteers to be a persuader at a highway junction. Other participants did not make an effort to achieve their aspirations and attributed this to practical constraints. The participants were in areas with community organizations, and the government departments were actively assembling people who were doing what the participants wanted to do. If they wanted to take the initiative to contact community organizations and government departments and apply to participate in the work, they should have been able to participate in the things they wanted to do. This shows that their ability to solve problems remains to be improved.

## Discussion

### Sophomore Nursing Students Have a Positive Attitude Toward COVID-19 Epidemic Events and Appropriate Practice, Which Is a Comforting Attitude

Participants felt a certain degree of panic about the epidemic. This emotion is a normal response of individuals to adverse events, which is consistent with the results of a large sample questionnaire survey of medical students (17). During the SARS epidemic in 2003, similar results were obtained, and 93.5% of medical students believe that a certain degree of psychological panic is a normal psychological response, which helps cope with the crisis (18). The participants' anxiety due to the inconvenience of life and academic stagnation caused by home isolation is also in line with the emotional state of the public (19). Participants had a clear and positive attitude toward the COVID-19 epidemic event.

Using existing knowledge and technology to contribute to the fight against the epidemic is worthy of recognition and praise. On the one hand, they are willing to respond to the call to stay home, and learn scientific ideas and methods of fighting the disease. They are also willing to do more to combat the epidemic, including devoting themselves to community publicity and disinfection work or participating in the screening and triage work of the floating population.

### Strengthening Evidence-Based Practice Ability in Nursing Teaching Helps to Improve Nursing Students Ability to Deal With New Public Health Events

Evidence-based practice competencies are important competencies that nurses must possess. The emergence of new public health events is often accompanied by a large amount of noisy information, and it is necessary to obtain useful scientific information carefully and discriminately to guide decision-making. Nurses with strong evidence-based practice ability can quickly select the strongest evidence from existing information to formulate new strategies and measures when faced with new things, to facilitate a faster and more scientific response to new public health events, especially emergencies.

Although evidence-based practice is becoming more and more critical in nursing work, undergraduate nursing students are not interested in it and lack the corresponding knowledge and skills. Researchers proposed that cultivating evidence-based practice competence in undergraduate nursing courses should be a priority in nursing education (20). This research also shows the urgency to cultivate evidence-based practice in undergraduate nursing education. According to the actual situation of nursing education in China, the training of evidence-based practice ability for undergraduate nursing students can be carried out from the following two aspects: (1) improve the curriculum and evidence-based teaching conditions; and (2) use a variety of teaching strategies such as problem-based teaching and scenario-based teaching to guide students to filter, analyze, evaluate, and use information (21). According to the results of this study, in future nursing courses, more emphasis should be placed on the concept of evidence-based practice, and students should be guided to apply evidence-based methods to evaluate the level of information and make scientific and practical decisions.

### Nursing Professional Education Must Cultivate Problem-Solving Ability During the Whole Process

Problem-solving ability refers to the nursing students identifying problems, setting a goal, making a choice, and implementing the problem-solving strategies (22). Problem-solving ability is a core competency of undergraduate nursing students (23). It is crucial in nursing education because the key challenge for undergraduate and graduate nursing schools is to perform nursing tasks that enable students to safely and effectively solve problems in a complex and changing healthcare environment (24). Previous studies in China have shown that nursing students have low problem-solving ability (25, 26). Educators attach great importance to the cultivation of senior nursing students' ability to solve nursing problems and guide nursing students to solve problems in clinical situations during the last year's internships before their graduations. However, the cultivation of problem-solving ability is not accomplished in one move, and attention should be paid to the cultivation of this ability among nursing students throughout the school period.

This study suggests that the current undergraduate nursing education should cultivate the problem-solving ability during the early years of nursing school. On the other hand, even in the stage of unfinished nursing undergraduate education, when a large-scale public health event occurs, nursing students should participate in part of the anti-epidemic work as students, and they will also face various emergencies that require them to have strong problem-solving skills. The COVID-19 epidemic has a strong shock to second-year undergraduate nursing students who have been exposed to the incident. Its origin, outbreak, epidemic process, and formulation and implementation of prevention and control plans and measures include many problems that need to be resolved, which are good teaching examples for improving problem-solving awareness and ability.

### Limitations

In this study, interviews were conducted when everyone had to be isolated at home. The outbreak of the COVID-19 epidemic was very sudden, and it happened to be during the winter vacation of all schools in mainland China. It was not easy to contact teachers and students from other schools for multicenter research. Therefore, the participants selected in this study were limited to a specific medical university in Chongqing. The latter have a teacher-student relationship with researchers and can communicate promptly, which cannot fully represent the situation of second-year undergraduate nursing students in China. Additionally, since we could not meet in person, we could not conduct face-to-face interviews. Furthermore, most of the respondents were reluctant to accept video interviews during their stay at home for reasons such as personal privacy and equipment conditions. Therefore, we conducted telephone interviews. These factors may have some influence on the research. However, the results of this study can still provide some clues for nursing education in China. For example, more attention should be paid to the cultivation of evidence-based practice ability and problem-solving ability.

## Conclusion

During the early stage of the COVID-19 epidemic, sophomore nursing students who have completed public health courses have a positive attitude toward large-scale public health emergencies. However, there is still a lack of knowledge of infectious diseases and epidemiology, evidence-based practice ability, and problem-solving skills. There are also deficiencies in nursing ability, suggesting that when entering the professional nursing course, the knowledge of infectious diseases and epidemiology should be further strengthened, and the training of evidence-based practice ability and problem-solving ability should be strengthened to improve the ability of nursing undergraduates to cope with large-scale public emergency after graduation.

## Data Availability Statement

The original contributions presented in the study are included in the article/supplementary material, further inquiries can be directed to the corresponding author.

## Ethics Statement

The studies involving human participants were reviewed and approved by Ethical Committee of Army Medical University. The patients/participants provided their written informed consent to participate in this study.

## Author Contributions

HZ and RZ contributed to the conception and design of the study, organized the database, performed data analysis, and wrote the first draft of the manuscript. YY designed the study, wrote sections of the manuscript, and reviewed the article. All authors contributed to the revision of the manuscript, read, and approved the submitted version.

## Funding

This study was supported by the Chongqing Higher Education Teaching Reform Research Project (No. 213481).

## Conflict of Interest

The authors declare that the research was conducted in the absence of any commercial or financial relationships that could be construed as a potential conflict of interest.

## Publisher's Note

All claims expressed in this article are solely those of the authors and do not necessarily represent those of their affiliated organizations, or those of the publisher, the editors and the reviewers. Any product that may be evaluated in this article, or claim that may be made by its manufacturer, is not guaranteed or endorsed by the publisher.
